# Effect of Multidirectional Forging and Aging Treatment on Wear Properties of ZK61 Alloy

**DOI:** 10.3390/ma17020523

**Published:** 2024-01-22

**Authors:** Xuhui Zhang, Jian Xu, Wenyu He, Jingjing Jia

**Affiliations:** 1 College of Materials Science and Engineering, North University of China, Taiyuan 030051, China; 2 College of Mechanical and Electrical Engineering, North University of China, Taiyuan 030051, China

**Keywords:** ZK61 alloy, MDF, grain refinement, wear resistance

## Abstract

This study investigated the effects of multidirectional forging (MDF) and aging treatments on the wear properties of ZK61 magnesium alloy. Dry sliding wear tests were performed on homogenized, MDF, and aged samples using a friction wear machine to analyze the surface morphology by scanning electron microscopy (SEM) and white light interferometry, as well as the hardness and tensile mechanical properties. The ZK61 magnesium alloy has higher sliding wear properties after MDF due to higher strength, hardness, and work hardening. Grain refinement affects the wear resistance of the material, but aging increases the hardness and tensile strength and decreases the wear resistance.

## 1. Introduction

Ultrafine-grained materials (UFGs) have better comprehensive properties than traditional coarse-grained materials (CGs) owing to their small grain size and large grain boundary area [1,2]. Therefore, UFG materials have received increasingly extensive attention from scholars in recent years and become one of the research hotspots in the field of metal structure materials. Traditional CG materials increase the density of internal defects (including point defects and surface defects) through severe plastic deformation (SPD) to obtain a higher proportion of large-angle grain boundary structures, thereby exhibiting superior comprehensive properties (including mechanical properties and physical and chemical properties) [3,4,5,6,7]. This is a popular research method for developing new materials and optimizing traditional materials.

Compared with other preparation technologies, SPD has the advantages of simple process equipment, wide application range, and good grain refinement effect, and can ensure that the size and shape of the material before and after deformation do not change. In general, SPD exerts external forces on the material so that the strain accumulates inside the material and the grains are broken and refined to obtain the UFG material with improved hardness, higher strength, and a lower friction coefficient, thereby improving wear resistance. Some researchers believe that the wear resistance of metals does not entirely depend on the size of the grains due to the variety of factors affecting the wear characteristics of materials [8,9].

Frictional wear is a complex systematic behavior that needs to take into account the influence of the friction partner, the lubricant, the environment, and the operating parameters [10]. It is generally recognized that there is a relationship between the microstructure (e.g., grain and precipitation phase size, morphology, and distribution) and mechanical properties (e.g., strength, hardness, plasticity, and grain boundary diffusion capacity) of UFG materials and their friction and wear properties. As the lightest metal structures and functional materials, magnesium and magnesium alloys are often used in aerospace, transportation, electronic communication equipment, and other important components due to their low density, high specific strength and specific stiffness, excellent heat resistance and toughness, and easy recycling [11,12]. Wear is a phenomenon in which the surface of a workpiece produces a constant loss of material in the process of relative sliding due to the presence of friction. Friction and wear will reduce the size of magnesium alloy parts, eventually leading to serious structural failure. However, wear is complex and influenced by several factors, so it is difficult to explore its mechanism only through macroscopic observation. Li et al. [13] improved the wear resistance of Cu–Zr alloys by obtaining the UFG structure by ECAP. Chegini et al. [14] studied the friction and wear characteristics of Al-7075 with different passes of ECAP at room temperature, showing that the lower the friction coefficient of the alloy with more passes, the better the wear resistance. However, there are few studies on the friction and wear properties of SPD ZK61 magnesium alloy. In this work, the ZK61 mg alloy was treated with multidirectional forging (MDF) and aging, and then its microstructure, hardness, strength, ductility, friction coefficient, and friction and wear characteristics were evaluated. On this basis, the effects of the microstructure, mechanical properties, and friction coefficient of the ZK61 magnesium alloy with a fine grain structure on friction and wear characteristics under dry sliding conditions were discussed in detail.

## 2. Experimental Procedure

**Figure 1:**
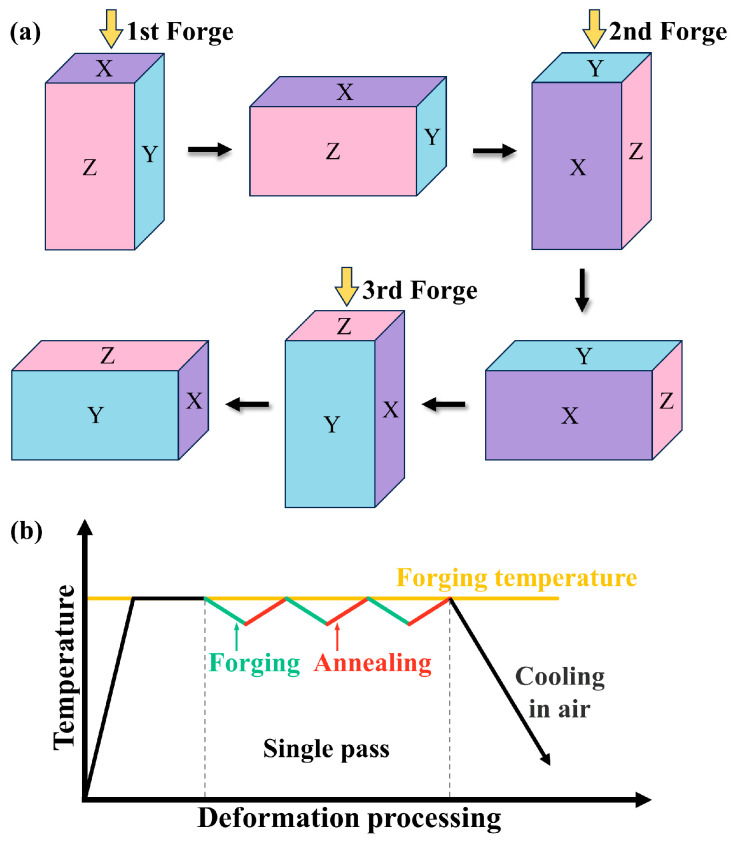
(**a**) Schematic illustration and (**b**) flow diagram of the MDF process.

The experimental alloy was a commercially available rolled ZK61 sheet, and the chemical composition of the alloy is shown in Table 1. The sample was water-quenched after homogenization at 400 °C for 50 h, then cut into 100 mm × 60 mm × 40 mm billets before proceeding to MDF. First, the billets were heated to 400 °C in a furnace for 2 h. Since heat is consumed when the mold is installed, the mold temperature is higher than the billet temperature, and the mold was kept at 450 °C for 6 h. As shown in Figure 1a, the initial forging direction is parallel to the extrusion direction, and the loading direction changes by 90° per pass (i.e., X to Y to Z to X ···) between the continuous forging passes. When the deformation in the X, Y, and Z directions is completed, it is known as a complete deformation pass of MDF, with a total of two passes performed. There were no significant changes in the dimensions of the blanks after MDF, and the samples were cooled to room temperature in air, as shown in Figure 1b. The MDF samples were aged at 150 °C for 36 h before the microstructure and mechanical properties after different treatments were analyzed.

**Figure 2:**
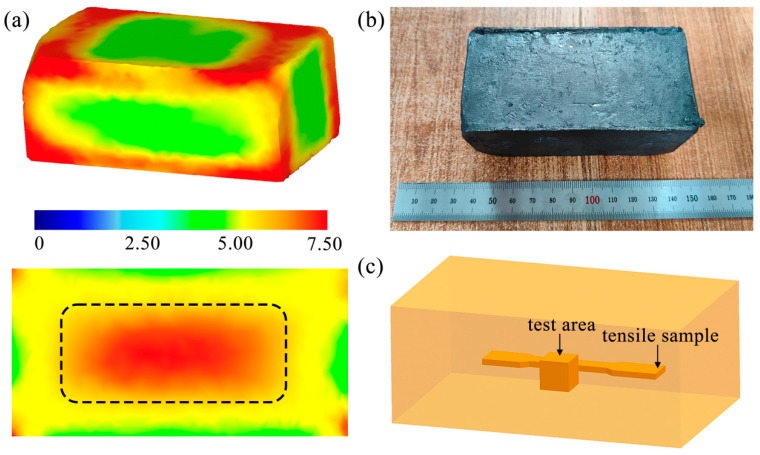
(**a**) Equivalent strain distribution diagram and section view of multidirectional forgings, (**b**) macroscopic morphology of the rolled ZK61 sheet after two MDF passes, and (**c**) schematic diagram of sample observation direction and position.

Finite element simulation technology was applied to obtain the ideal performance of the finished sample, as shown in Figure 2a. The DEFORM-3D (v11.0) finite element software was used to simulate and analyze the forging process, which simulates the stress and strain of the metal material during forging. The equivalent strain of the two-pass forging in Figure 2a shows that the strain in the central area of the forging is the largest with a maximum of 7.5 (in the black dashed box) and is distributed from the center to the four corners. The closer to the center, the higher the degree of grain refinement, and the larger the equivalent strain, the smaller the grain size. The samples after MDF have no surface defects, as shown in Figure 2b. Therefore, wear test samples and tensile sheets were obtained in the central region of the MDF sample, as shown in Figure 2c.

The hardness values of the samples under different treatment conditions were measured by a Vickers hardness tester. In order to make the measurement results more accurate, 15 points were marked on each sample, and the maximum and minimum values were removed, and their average values were taken as the final hardness values. During the test, the load was 150 g and the holding time was 15 s. Then the samples were stretched at room temperature by a stretching machine. The tensile strain rate was 1 × 10^−3^ s^−1^, and the tensile direction was parallel to the deformed sheet. More than three repetitions of the experiment were performed to ensure the accuracy of the results.

Three samples with different treatments were subjected to friction and wear experiments. The friction and wear experiments were carried out on an MFT-4000 high-speed reciprocating friction and wear tester, and the friction was in the form of dry sliding friction at room temperature. The material was prepared into a square wear sample with a size of 10 mm × 10 mm × 10 mm by wire cutting. The wear surface of the sample was uniformly ground by 7000 # Sic and finally polished to the surface without obvious scratches. The friction pair material was a GCr15 steel ball. Before and after the wear experiment, the samples were ultrasonically cleaned in anhydrous ethanol. After drying, the mass of the sample was weighed using an electronic balance, and the mass loss before and after wear was calculated, that is, the wear amount (m). In this experiment, as shown in Table 2, the wear test was carried out at normal temperature and pressure. The load was 20 N, the friction speed was 50 mm/min, the reciprocating sliding distance was 5 mm, and the friction time of each sample lasted for 15 min. The friction coefficient was automatically recorded by a sliding wear tester.

The EBSD specimen was first mechanically polished, and then electrolytically polished in a solution (10% HClO_4_ + 90% C_2_H_5_OH) for 80 s at 15 V. Finally, the OIM7.2 software was used to analyze the EBSD data. The wear surface was observed by scanning electron microscopy (SEM, Hitachi SU5000, Hitachi, Shanghai, China).

## 3. Results

### 3.1. Microstructure and Mechanical Properties

**Figure 3:**
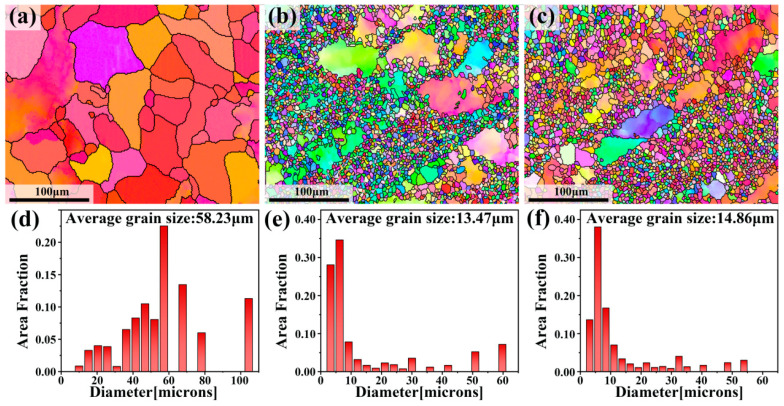
EBSD maps and corresponding grain size maps: (**a**,**d**) homogenized sample, (**b**,**e**) MDF sample, and (**c**,**f**) aged sample.

In order to further investigate the initial microstructure of the three samples, EBSD analysis was performed. Figure 3a–c show the EBSD maps of homogenized samples, MDF samples, and aged samples. It can be found that the homogenized samples had significantly finer grains after passing through two MDF passes. After the aging of the MDF sample, the grain size changed little because of the low aging temperature. Figure 3d–f show the grain size distribution maps of three samples. The average grain sizes of the homogenized sample, MDF sample, and aged sample were about 58.23, 13.47, and 14.86 μm, respectively. The maximum grain size of the homogenized sample can reach 104.48 μm. After MDF, the maximum grain size was reduced to 60.51 μm, and the average grain size of fine grains was 6.25 μm. The average grain size of the homogenized sample was reduced by 23% after MDF.

**Figure 4:**
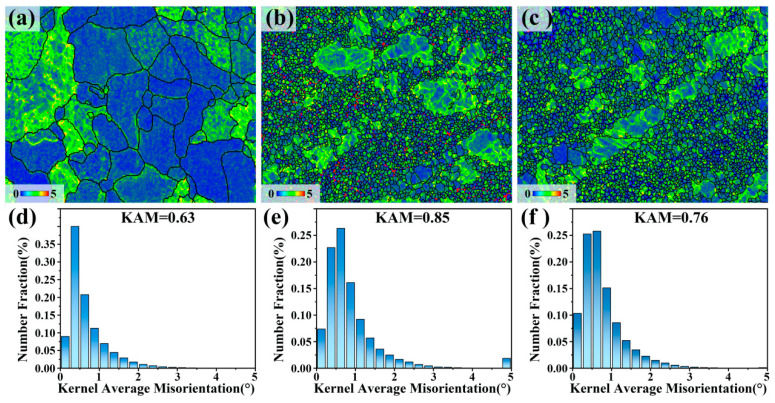
The kernel average misorientation (KAM) and corresponding KAM distribution: (**a**,**d**) homogenized sample, (**b**,**e**) MDF sample, and (**c**,**f**) aged sample.

Figure 4 shows the kernel average misorientation (KAM) map and the corresponding KAM distribution map of the three samples. The KAM map shows the strain degree and local dislocation density of the sample after the deformation process. Three different colors, blue, green, and red, are usually used to indicate dislocation density. The gradual increase in dislocation density is indicated by the color change from blue to green to red, which can also be used to indicate the concentration of local residual stress [15,16]. Figure 4a shows that there are many green areas in some CGs and their grain boundaries of the homogenized samples, but the distribution is very uneven. Figure 4b,c show that the green areas of the MDF sample and the aging sample are more widely and evenly distributed. In the MDF sample, there are red areas at some fine grain boundaries that are reduced after aging. It can be seen that the green area in the KAM map of the homogenized sample increases after MDF (from Figure 4a,b), indicating an increase in dislocation density. This phenomenon indicates that dislocations accumulate in the grains due to the generation of strain. Figure 4d–f show that the average KAM values of the three samples are 0.68, 0.89, and 0.76, respectively. The precipitated phases and macrotexture of the samples after MDF and aging treatment are shown in Figures S1 and S2.

**Figure 5:**
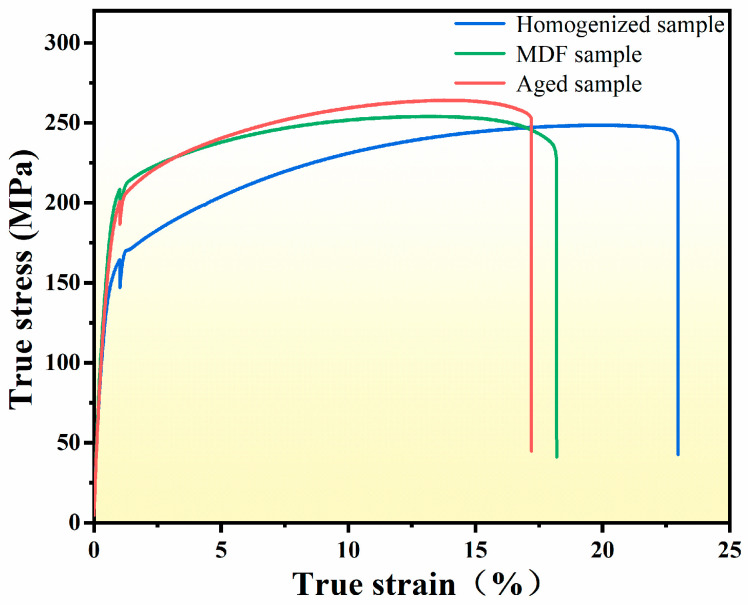
The stress–strain curves of the three samples.

The hardnesses obtained by hardness testing for homogenized samples, MDF samples, and aged samples were 57.57, 65.48, and 67.85 HV, respectively. Figure 5 shows the engineering stress–strain curves for the three samples. The tensile strength and ductility of the homogenized sample were 248.63 MPa and 24.9%, respectively. After MDF, the tensile strength of the sample increased to 254.11 MPa, but the ductility decreased to 20.6%. The MDF sample achieved a tensile strength of 264.18 MPa and an elongation of 19.4% by aging.

**Figure 6:**
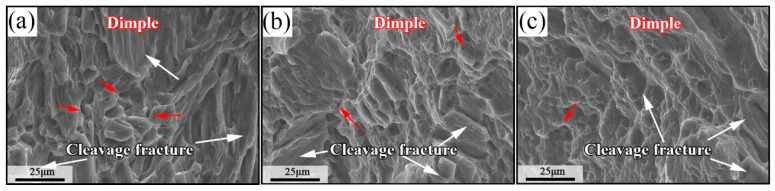
Sample fracture morphology: (**a**) homogenized sample, (**b**) MDF sample, and (**c**) aged sample.

Figure 6 shows that the three samples are mixed fracture, including dimples and cleavage platforms. By comparison, there are relatively more dimples in the homogenized sample, and the depth of the dimples is deeper. The other two samples have fewer dimples and a shallower depth. This will make the homogenized sample have a good elongation. In addition, it was found that the cleavage planes of the three samples are relatively close, so the influence on the strength of the sample is small.

### 3.2. Friction and Wear Mechanism

**Figure 7:**
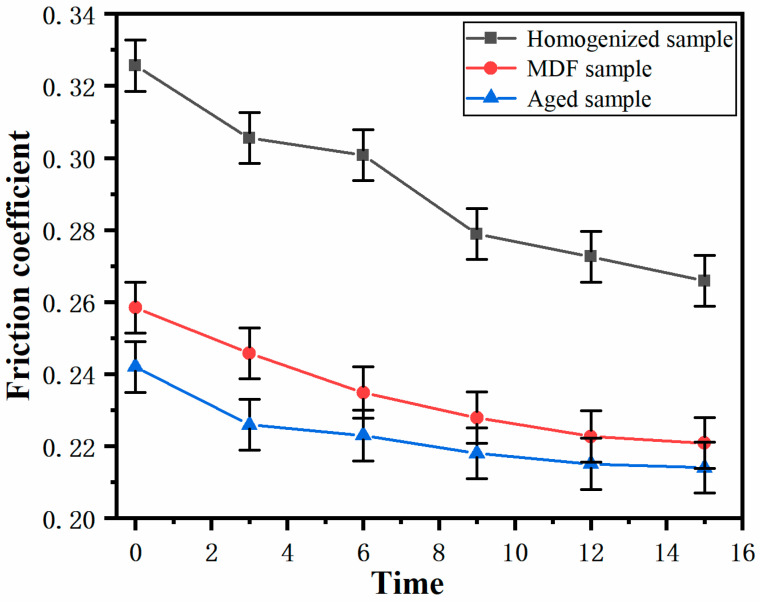
The friction coefficient of the three samples changes with the test time.

The friction coefficient has a decisive effect on the wear resistance of a mechanical structure [17,18]. Figure 7 shows the variation trend of the average friction coefficient of the homogenized sample, the MDF sample, and the aged sample under continuous sliding friction. As can be seen from the figure, among the three groups of samples, the average friction coefficient of the homogenized sample is the largest, followed by the MDF sample, and the aged sample has the smallest average coefficient of friction. It may be due to the grain crushing and refinement of the sample after MDF treatment, which improves the wear performance of the sample, and during the wear process, due to the severe plastic deformation caused by MDF, the plastic strength decreases accordingly, resulting in a decrease in the wear marks of the sample, especially the depth. As a result, the friction coefficient of the MDF sample becomes smaller. The hardness of MDF samples was further improved by aging treatment. The higher the hardness of the sample is, the lighter the friction pair falls into the surface of the sample. The friction coefficient decreases due to the decrease in the actual contact area and the molecular force between the sample surface. Figure 7 shows that with the increase in time, the average friction coefficient of homogenized samples, MDF samples, and aged samples is large from the beginning and then gradually decreases. At the beginning, the larger friction tangential force hinders the sliding of the friction pair, so the friction coefficient rises to the maximum at the beginning. When the two friction pairs have relative sliding wear, the running-in stage is first carried out. When the wear surface is gradually flattened and the contact area increases, it will enter a normal and stable sliding so that the friction coefficient begins to decrease and remain in a stable state. This is the main reason for the above phenomenon.

Table 3 shows that among three samples, the wear resistance of the homogenized sample is the lowest, only 62.5 g^−1^. The wear resistances of the multidirectional forging and aging samples have been greatly improved, which are 142.8 and 111.1 g^−1^, respectively, which are about 128.5% and 77.8% higher than that of the homogenized samples. Therefore, it shows that the wear resistance of the UFG materials obtained by SPD is better than that of coarse-grained materials, and it also shows that multidirectional forging has more obvious advantages under dry sliding wear conditions, with the ability to make alloys significantly more resistant to sliding wear.

The wear mechanism can be divided into adhesive wear, oxidation wear, abrasive wear, and so on [19,20]. Adhesive wear [21] is primarily caused by the contact between two solid surfaces. Due to the uneven surface, it is actually the contact between the asperities. Under the action of relative velocity and pressure, plastic deformation occurs between the contact asperities, and the temperature of the friction surface increases. In severe cases, the metal will melt and the contact point will adhere. At the microscopic level, wear patterns such as scratches, abrasions, or material transfer on the specimen surface can be observed [22]. Oxidation wear is the most common form of wear, which occurs in any friction process. When a protrusion on one side of a friction specimen slides against the other side, plastic deformation occurs while oxygen diffuses into the deformed layer to form an oxide film. This oxide film may flake off when it encounters the second projection so that the newly exposed surface is oxidized again, and the process keeps cycling. From the microscopic point of view, after frictional wear of the sample, wear surface granular abrasive debris or black oxide bands and other wear patterns can be observed. Abrasive wear [23] is the most common form of mechanical wear, a basic type of wear. Abrasive wear is the loss or material loss caused by the extrusion and movement of hard particles or hard asperities on the surface of the solid surface during the friction process. At the microscopic level, it can be seen that the abrasive is pressed into the friction surface to produce indentation, and the surface of the plastic material is squeezed out of the wear morphology, such as layered or scaly spalling debris.

**Figure 8:**
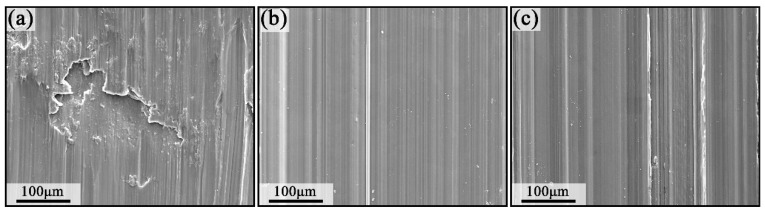
The microstructure of the wear scar of the sample: (**a**) homogenized sample, (**b**) MDF sample, and (**c**) aged sample.

Figure 8a shows the microscopic morphology of the wear scar of the homogenized sample. There are obvious furrows, obvious scratches, and scratches on the surface of the sample, as well as delamination and a small amount of granular wear debris on the edge of the sample, indicating that abrasive wear and adhesive wear occur during the friction and wear process of the homogenized sample. Figure 8b shows the microscopic morphology of the abrasion marks on the MDF samples, which showed obvious furrows, slight abrasions, and black oxidized bands on the surface of the samples after frictional wear, indicating that abrasive wear, adhesive wear, and oxidative wear occurred during the frictional wear process of the samples. Figure 8c shows the microscopic morphology of the abrasion marks of the aged sample, with obvious furrows on the surface of the sample and a few white bright-colored abrasive flakes, which denotes that abrasive wear, as well as adhesive wear, occurs in the frictional wear process of the aged sample.

**Figure 9:**
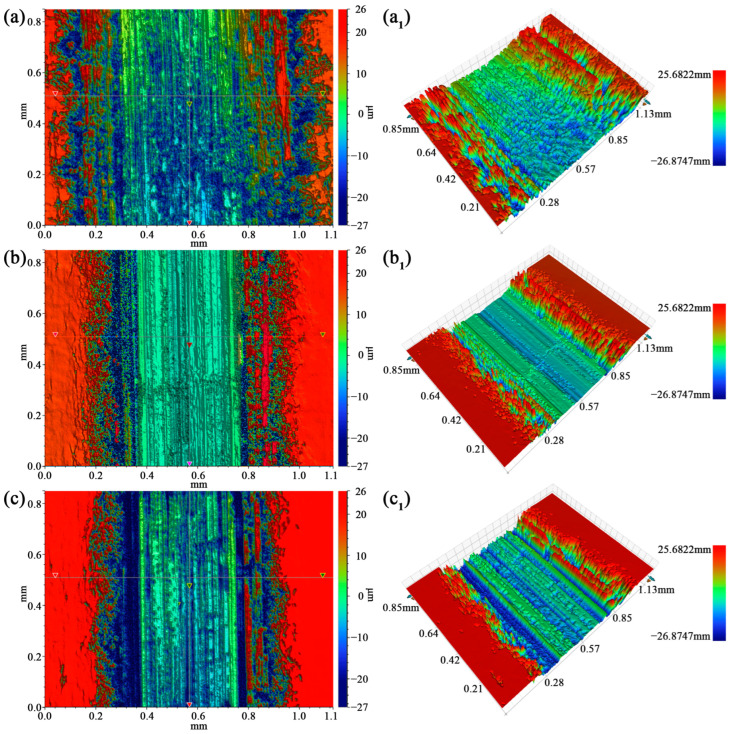
White light interferogram maps of three samples: (**a**,**a_1_**) homogenized sample, (**b**,**b_1_**) MDF sample, and (**c**,**c_1_**) aged sample.

Figure 9 shows the three-dimensional morphology of the samples in different states measured by a white light interferometer. The data show that the wear scar width of the three samples is not much different, and the wear scar depth has different degrees of difference. The homogenized sample has the greatest depth of abrasion marks, followed by the MDF sample, and the aging sample has the least depth of abrasion marks. The wear scar depths of the MDF and aged samples is not much different. The three-dimensional topography was analyzed and compared with the width and depth of the wear marks of the homogenized sample. After the MDF treatment of the sample, it can be clearly seen that the width and depth of the wear marks of the sample were significantly reduced during the friction and wear process. This may be because the sample was subjected to MDF treatment, and the grains were broken and refined under severe plastic deformation, and then the UFG materials were obtained, so that the friction and wear properties were improved. The width and depth of the abrasion marks of the aged sample were not much different from those of the MDF sample, which may be due to the fact that the aging treatment reduced the dislocation density and residual stress of the samples, improved the plasticity of the samples, and therefore improved the wear performance of the samples.

## 4. Discussion

The microstructure of materials is closely related to their mechanical properties. MDF greatly improves its hardness and strength, which is mainly due to the fact that SPD occurs in the MDF process [24,25], which can significantly refine the grain structure. On the one hand, the number of grain boundaries increases with grain refinement; on the other hand, the density of dislocations within the material also increases dramatically due to the increasing amount of deformation, so the comprehensive effect of the grain refinement mechanism and dislocation theory enhances the degree of work hardening and leads to a substantial increase in the hardness of the material. MDF, as one of the techniques of SPD, can lead to a significant refinement of the grain organization and an increase in its strength and hardness, in line with the well-known Hall–Petch formula [26,27,28]. However, like other strong plastic deformation processes, it shows a sharp decline in elongation, which is a typical feature of deformed materials [29,30]. The reason for this, according to Valiev et al. [31], is that the plastic deformation mechanisms and dislocation motion of the material do not work due to ultrafine grains or ultrafine organization accompanied by an increased number of grain boundaries.

The number of grain boundaries increases due to grain refinement, and the increase in dislocation density causes dislocations to cross and tangle with each other, making dislocation movement at the grain boundaries more hindered, resulting in dislocation plugging and increasing strength and hardness of material. According to theories such as the Hall–Petch formula,

(1) $$\sigma_{y}=\sigma_{0}+kd^{-\frac{1}{2}}$$

where $\sigma_{0}$ is the frictional resistance to dislocation movement within the grain, *k* is a constant, and *d* is the average grain size. The smaller the grain size of metal materials, the higher the strength and hardness, which has been verified by a large number of studies.

The ZK61 homogenized alloy is subjected to strong plastic deformation by multidirectional forging [32,33,34], which results in significant grain refinement and an increase in density of grain boundaries. With the increasing amount of deformation, the dislocation density increases and entanglement occurs, and its deformation resistance also increases, thus substantially increasing the strength and hardness of the material. In general, the factors that affect the wear of a material include its properties such as hardness, strength, ductility, and work hardening [35]. Based on the fact that the wear loss and wear rate decrease with the increase in the hardness of the material, the main reason for the improvement of the wear resistance of the material can be explained by the following Archard formula [36]:

(2) $$Q=\frac{KPL}{H}$$

where *Q* represents the wear volume, *P* is the wear load, *L* is the wear stroke, *H* is the Vickers hardness, and *K* is a constant related to the wear coefficient.

The wear volume *Q* is related to the density ρ of the material, which can be expressed by the following formula:

(3) $$Q=\frac{W}{\rho }$$

where *W* represents the amount of wear (mg). Therefore, the Archard formula can also be written as follows:

(4) $$\frac{W}{\rho }=\frac{KPL}{H}$$

Formula (4) shows that when the constant K and the wear load are constant, the wear amount W is proportional to the wear stroke L and inversely proportional to the hardness of the wear material. In this experiment, the amount of material wear decreases with the increase in hardness, and its wear properties are in accordance with the Archard formula, which is favorable to the increase in wear resistance of ZK61 magnesium alloy after MDF.

**Figure 10:**
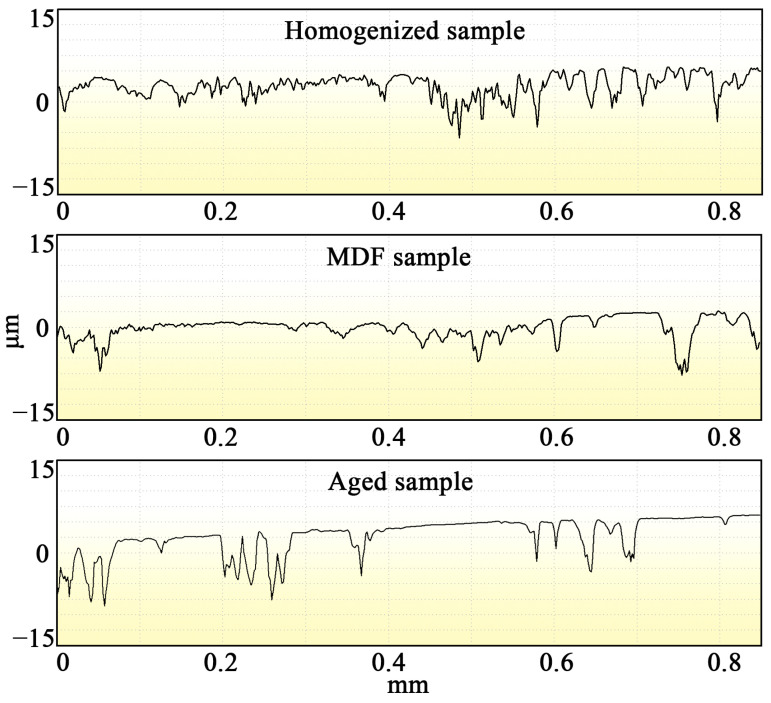
Cross section of the three samples after wear.

Surface roughness (SR) refers to the roughness of the machined surface with a small spacing and a small peak valley [37,38]. The double-peak or double-valley spacing is very small (less than 1 mm), which is a microgeometry error. With the decrease in SR, the smoothness increases. In actual production, this is due to the use of different processes and materials, as well as some other factors, resulting in changes in SR. Differences in processing methods and workpiece materials result in traces of varying depths, densities, shapes, and textures on the surfaces being processed. The SR of mechanical parts is an important indicator of their wear resistance. Figure 10 shows that the fluctuation range of the broken line of the homogenized sample is larger than that of the MDF and aged samples. The average surface roughnesses of the homogenized, MDF, and aged samples are 15.89, 8.56, and 10.23 μm, respectively. The wear surface of the MDF sample is less rough than those of the homogenized and the aged samples, indicating that the surface is smoother [39,40,41].

In summary, existing studies have found that the relationship between SPD material hardness and work hardening and wear is not comprehensive. Friction and wear are not determined by the material itself, but by the friction system. Therefore, it is necessary to conduct a comprehensive and in-depth study on the tribological properties of SPD materials.

## 5. Conclusions

(1) The average friction coefficient of the homogenized sample is the largest, followed by the MDF and the aged samples. The average friction coefficients of all three treatment states reach a maximum at the beginning and enter the steady friction state so that the coefficient of friction begins to decrease and remains in the steady state.
(2) The wear mechanism of the homogenized and aged samples is mainly abrasive and adhesive wear, whereas the wear mechanism of the MDF sample is mainly abrasive, adhesive, and oxidative wear.
(3) Fine-grained samples were obtained after MDF treatment. The samples after MDF and aging treatments have improved sliding wear properties and hardness and sufficient work hardening. The wear resistance of the MDF sample is higher compared with those of the homogenized and the aged samples.
(4) MDF is valuable for the improvement of the wear resistance of ZK61 magnesium alloy. Improving wear resistance helps to broaden the use of ZK61 magnesium alloys in a variety of applications, especially where high wear resistance is required. This also provides inspiration and reference for the study of wear resistance of other metallic materials.

## Figures and Tables

**Table 1:** Actual composition of the ZK61 alloy (wt.%).

Alloy	Zn	Zr	Mg
ZK61	6.02	0.56	Bal.

**Table 2:** Experimental parameters of friction and wear.

Load	20 N
Speed	50 mm/min
Reciprocating sliding distance	5 mm
Time	15 min

**Table 3:** Average wear resistance after sliding wear.

Samples of Different Treatment States	Average Wear Resistance (g^−1^)
Homogenized sample	62.5
MDF sample	142.8
Aged sample	111.1

## Data Availability

The data presented in this study are available on request from the corresponding author.

## Supplementary Materials

The following supporting information can be downloaded at https://www.mdpi.com/article/10.3390/ma17020523/s1: Figure S1: X-ray diffraction patterns of different samples; Figure S2: The macrotexture of three samples: (a) homogenized sample, (b) MDF sample, and (c) aged sample.
