# Supplementary material for: Effect of Multidirectional Forging and Aging Treatment on Wear Properties of ZK61 Alloy
Source: Materials (Basel). 2024 Jan 22;17(2):523. doi: 10.3390/ma17020523 (PMC10817632; doi:10.3390/ma17020523)
Supplement: Supplementary file 1 [file materials-17-00523-s001.zip › materials-2802464-supplementary.pdf]

materials MDPI

Supplementary Material

Effect of Multi-Directional Forging and Aging Treatment on Wear Properties of ZK61 Alloy

Xuhui Zhang [1], Jian Xu [1,*], Wenyu He [1] and Jingjing Jia [2]

[1] College of Materials Science and Engineering, North University of China, Taiyuan 030051, China
[2] College of Mechanical and Electrical Engineering, North University of China, Taiyuan 030051, China
* Correspondence：20160608@nuc.edu.cn

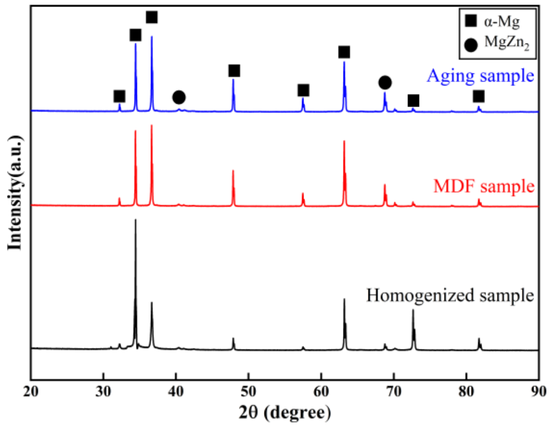

Figure S1 X-ray diffraction patterns of different samples.

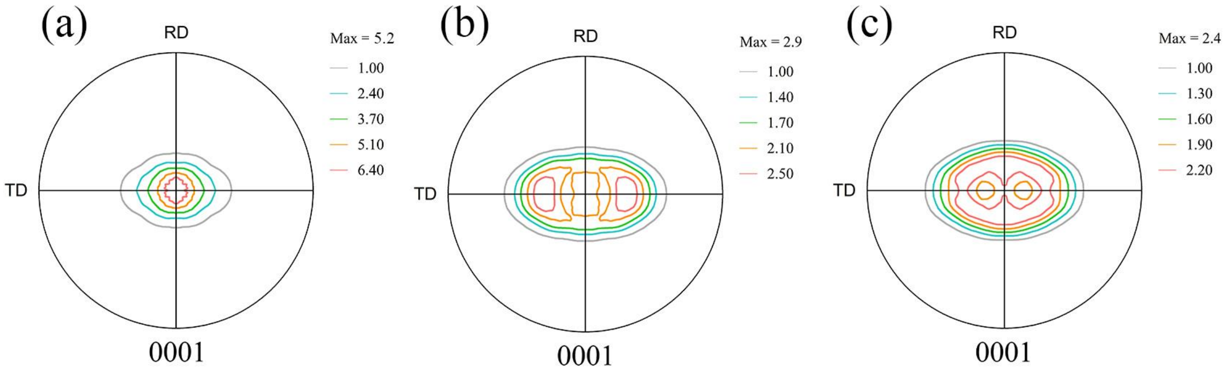

Figure S2 The macro-texture of three samples: (a) homogenized sample; (b) MDF sample; (c) aged sample

Figure S1 shows the X-ray diffraction (XRD) patterns of the experimental sample under the different treatment methods. It is obvious to find that the samples are consisting of Mg matrix and $MgZn_2$ phases. Because there is no new precipitation in the three samples, it does not impact the tribological properties. Figure S2 shows the macro-texture of three samples, it is found that the change of texture is relatively small. Therefore, the influence of macro-texture on its tribological properties is not analyzed.
